# Silver diamine fluoride, atraumatic restorations, and oral health-related quality of life in children aged 5–13 years: results from the CariedAway school-based cluster randomized trial

**DOI:** 10.1186/s12903-022-02159-5

**Published:** 2022-04-12

**Authors:** Ryan Richard Ruff, Tamarinda J. Barry Godín, Topaz Murray Small, Richard Niederman

**Affiliations:** Department of Epidemiology & Health Promotion, New York University College of Dentistry, New York, USA

## Abstract

**Objective:**

Silver diamine fluoride (SDF) is a non-surgical treatment for the arrest and prevention of dental caries that results in irreversible black staining of dental decay. The objective of this study was to evaluate the short-term impact of SDF treatment on oral health-related quality of life (OHRQoL) relative to a standard package of glass ionomer sealants and atraumatic restorative treatment (ART) in children aged 5–13 years.

**Methods:**

CariedAway is a pragmatic, longitudinal, cluster-randomized non-inferiority trial of non-surgical interventions for caries. Secondary study outcomes included OHRQoL and academic performance. Oral health-related quality of life was measured at each study visit using the Child Oral Health Impact Profile. Change in OHRQoL was assessed using linear regression and non-inferiority was determined using *t* tests.

**Results:**

160 children with an average age of 8.7 years completed quality of life assessments. Untreated decay at baseline (approximately 25%) was associated with significantly worse OHRQoL and treatment in both groups resulted in incremental improvement: children receiving SDF improved their OHRQoL scores from 16.44 (SD = 11.12) to 14.62 (SD = 11.90), and those receiving traditional sealants and atraumatic restorations slightly improved from 16.65 (SD = 10.56) to 16.47 (SD = 11.09). Quality of life in children receiving silver diamine fluoride was non-inferior to those receiving sealants and ART at least 6 months post-treatment (mean difference = 1.85, 95% CI = − 2.10, 5.80), and change in OHRQoL did not depend on the severity of baseline decay.

**Conclusions:**

OHRQoL is related to untreated dental caries, and observed changes following SDF treatment were non-inferior relative to standard preventive therapies.

## Introduction

Dental caries is the most prevalent childhood disease in the world [1], found across all age groups and most prominent among low-income populations [2]. Untreated caries has been shown to develop into pain and systemic infection, potentially resulting in functional and/or psychosocial impairment [3]. Much of the disproportionate burden of disease amongst vulnerable groups, such as low-income and minority populations, is due to lower accessibility and utilization of traditional dental services [4–6]. As a result, the use of non-surgical treatments such as silver diamine fluoride (SDF) is increasing. Silver diamine fluoride is a noninvasive method to prevent and arrest caries that can be efficiently applied in community settings [7–9], but results in permanent black staining of dental decay and could stain sound tooth structure.

Oral health-related quality of life (OHRQoL) is a multidimensional construct consisting of subjective evaluations of oral health, functional well-being, emotional well-being, satisfaction with care, and sense of self [10]. Caries may have a negative impact on oral health-related quality of life in preschool children [11], children aged 3–12 years and adults [12–14]. High caries experience [15] and untreated caries [16] both exhibit reduced OHRQoL, regardless of measurement used [17, 18]. Despite a high oral disease burden [19], research on quality of life and caries in black and Hispanic/Latino populations is limited [20, 21]; evidence on silver diamine fluoride and quality of life presents conflicting results with treatment shown to either improve or have no effect on OHRQoL in children [22–27]; and the impact on OHRQoL comparing SDF to atraumatic restorative treatment is unclear [24, 27].

CariedAway is a randomized controlled trial of non-surgical interventions for the prevention and treatment of caries in children aged 5–13 years [28], specifically silver diamine fluoride, sealants, and atraumatic restorative treatment. The CariedAway study also aims to evaluate the effects of treatment on quality of life, academic performance, and school attendance. The objectives of this paper are to assess (1) the associations between oral health-related quality of life and dental caries and (2) the short term effects of non-surgical treatment for caries on oral health-related quality of life.

## Methods

Ethical approval for the CariedAway clinical trial was obtained from the New York University School of Medicine Institutional Review Board (i17-00578). A previously published trial protocol contains additional study-related information [28] and the trial is registered at www.clinicaltrials.gov (#NCT03442309). Preliminary clinical results are forthcoming.

### Design

CariedAway is a longitudinal, cluster-randomized, single-blind, pragmatic trial with the primary objective of evaluating the non-inferiority of non-surgical treatments for dental caries. Any school in New York City with a student population of at least 80% receiving free or reduced lunch and at least 50% Hispanic/Latino or black was eligible to participate in the study. School-level exclusion beyond race/ethnicity and free/reduced lunch criteria included those with a preexisting school oral health program. All children in enrolled schools were provided informed consent, there were no inclusion criteria for child-level enrollment. Any subject with parental informed consent and child assent was randomly assigned to treatment and received care. Exclusion criteria for individual subjects included any child whose first language was anything other than English and children enrolled in special education classrooms. Treatment was provided in scheduled 6-month intervals. A total of 48 schools were enrolled in the CariedAway study at the time of this report.

### Interventions

Interventions included two separate packages of non-surgical treatments for dental caries: a “simple” combination of fluoride varnish (5% NaF, Colgate PreviDent) applied to all teeth and silver diamine fluoride (Elevate Oral Care Advantage Arrest 38%, 2.24 F-ion mg/dose) applied to all pits and fissures and asymptomatic cavitated lesions of bicuspids and molars, and a “complex” combination consisting of the same application of fluoride varnish, glass ionomer sealants applied to pits and fissures of bicuspids and molars, and use of atraumatic restorative treatment on all frank asymptomatic cavitated lesions (GC Fuji IX). For SDF application, tooth surfaces were cleaned and dried and a microbrush was used to transfer the solution to all pits and fissures on bicuspids or molar teeth and to all posterior, asymptomatic carious lesions for a minimum of 30 s, followed by air drying for a minimum of 60 s. Excess material was removed from teeth with a 2 $\times$ 2 gauze or cotton roll. Caries diagnosis followed the International Caries Detection and Assessment System (ICDAS) adapted criteria and the diagnostic and treatment protocol is previously described [28].

### Randomization

Enrolled schools were block randomized in a 1:1 allocation ratio.

**Table 1:** Sample demographics and clinical outcomes

Variable	Baseline sample (N = 1323)		Analytic sample (N = 160)	
	N/mean	%	N/mean	%
Sex				
Female	700	52.91	88	55
Ethnicity				
Hispanic	513	38.78	82	51.25
Non-Hispanic	105	7.94	15	9.38
N/A	705	53.29	63	39.38
Race				
Black	203	15.34	40	25
Asian	19	1.44	3	1.88
Multirace	34	2.57	4	2.5
White	56	4.23	3	1.88
Other	27	2.04	4	2.49
N/A	984	74.38	106	66.25
Clinical indicators				
Untreated decay	341	25.77	45	28.12
# Decayed teeth	0.49	1.09 (SD)	0.43	0.84 (SD)
Treatment group				
Simple (vs complex)	633	47.85	111	69.38

### Data collection

At each observation, study clinicians performed a full visual-tactile examination using a disposable mirror, disposable explorer, and head lamp in a portable dental chair or while using a lapboard. Teeth were assessed as being present or missing intraorally. Individual tooth surfaces were assessed as being intact/sound (ICDAS II codes 0–4), sealed, restored, decayed (ICDAS II code 5–6), or arrested [caries]. Clinicians were standardized prior to observing subjects (see supplementary material for procedures). Following the oral examination and application of treatments, children were asked to complete the oral health-related quality of life survey. The clinician would then read each question aloud to subjects who would then note their answers on a provided tablet computer.

### Outcomes

Oral health-related quality of life was assessed using the Child Oral Health Impact Profile Short Form (COHIP-SF), consisting of 19 questions assessing oral health, functional well-being, socio-emotional well-being, school environment, and self-image domains [29, 30]. Lower scores indicated “better” quality of life. Examiners asked participants each COHIP-SF question (e.g., “Have you in the past 3 months had pain in your teeth or a toothache”), and children responded by touching an indicator on the tablet or communicating their answer (“Never,” “Almost never,” “Sometimes,” “Fairly often,” “Almost all of the time”) to the examiner. The COHIP-SF was designed for children aged 8 and up, and the average age of subjects in CariedAway completing the OHRQoL instrument was 8.7 years. Additionally, 95.7% of subjects were aged between 7 and 11 years at baseline and study pretesting suggested that children aged 7 years were capable of completing the instrument. A global quality of life indicator measuring perceived change from the previous observation was also used. For the global QoL question, participants were asked “On a scale of 0 to 100 where 0 is worst and 100 is best, how would you rate your quality of life?” Subjects were then shown a visual scale on a horizontal axis ranging from 0 to 100 in 10-point intervals, with “worst” at the zero point, “medium” at the 50th point, and “best” at the 100th point. Participants then tapped their answer at the point of the scale directly on the tablet. Not all children received the COHIP-SF instrument: in order to reduce the data collection time and maximize provision of clinical care, fifty percent of participants in the CariedAway trial were randomly assigned within each group at baseline to receive the quality of life assessment. Assigned children then received the same QoL instrument at every successive treatment.

### Covariates

Demographic information including age, sex, and race/ethnicity were obtained from informed consent documents or school records. A unique identification number maintained by the Office of School Health at the New York City Department Health and Mental Hygiene and New York City Department of Education was similarly used as the patient record number for this study.

### Statistical analysis

Analysis was restricted to only those subjects between the ages of 5 and 13 years at time of observation. Subjects were analyzed using intent to treat: any child who may have switched schools that was randomized to a different treatment arm was analyzed according to his or her original assignment. Baseline descriptive statistics for sociodemographic variables and COHIP scores were computed. The association between dental caries at baseline and initial quality of life was assessed using linear regression. Following baseline analyses, subjects were ordered sequentially by visit date and any child without two completed visits was removed from the analytic sample. Within-group differences for treatment groups used paired samples *t* tests. Post-treatment analysis of the caries-QoL association was assessed using linear regression, adjusting for treatment group, baseline COHIP scores, and demographic variables. The intraclass correlation coefficient for subjects nested within schools was estimated using an intercept-only mixed effects multilevel model. All analyses adjusted for the clustering effect of schools. The non-inferiority of SDF therapy compared to sealants/ART on oral health-related quality of life was determined by calculating the confidence interval for the difference in mean COHIP scores across treatment group. As per the original study protocol, a non-inferiority margin of ten was used.

### Impact of COVID-19

Although all study subjects randomly selected to complete oral health-related quality of life assessments at baseline were expected to complete follow-up data collection at each successive measurement period, the impact of COVID-19 required suspension of study procedures for approximately 18 months due to the shutdown of local school systems and subsequent prohibition of any school-based health programs from operating. As a result, analysis was restricted to those subjects who had completed their first 6 month follow-up assessment just prior to shutdown.

## Results

1323 subjects completed the quality of life assessment at baseline, 160 of which (12%) completed a second-follow up at least 6 months post-treatment with either silver diamine fluoride or sealants/ART, prior to study suspension due to the impact of SARS-CoV-2. The analytic sample (N = 160) was spread across 17 schools. The baseline sample was approximately 53% female and consisted of 39% Hispanic/Latino, 15% black, 4% white, and 1.4% Asian (Table 1). The overall prevalence of any untreated decay on any tooth (deciduous or permanent) was 25.8% with an average per-person number of decayed teeth of 0.45 (SD = 1.1). Baseline untreated decay prevalence was 25% in the simple group and 35% in the complex group (analytic sample). The average time between treatments was 189 days. The intraclass correlation was 0.0326.

**Table 2:** COHIP-SF scale and subscale scores, pre/post, by treatment group (means and standard deviations; N = 160)

Scale/subscale	SDF		Sealants + ART	
	Pre	Post	Pre	Post
Oral health	4.86 (3.31)	4.38 (3.66)	5.12 (3.55)	5.61 (3.26)
Functional well-being	2.88 (2.88)	2.08 (2.83)	2.24 (2.54)	2.63 (2.85)
Socio-emotional well-being	4.25 (4.77)	3.73 (4.84)	4.49 (5.16)	3.67 (5.69)
School environment	1.09 (1.51)	0.97 (1.52)	1.39 (2.14)	0.91 (1.71)
Self-image	3.36 (2.34)	3.46 (2.73)	3.41 (3.01)	3.63 (3.03)
COHIP (overall)	16.44 (11.12)	14.62 (11.90)	16.65 (10.56)	16.47 (11.09)

Children with untreated decay at baseline, irrespective of treatment assignment, scored significantly worse on oral health-related quality of life (B = 3.67, 95% CI = 2.15, 5.18, data not shown) than those with no untreated decay. Adjusting for differences in race and ethnicity, each decayed tooth was associated with 1.34 (95% CI = 0.69, 1.99, data not shown) point increase in COHIP scores, where higher scores indicate worse OHRQoL. There were no baseline differences in OHRQoL by treatment group (B = − 0.61, 95% CI = − 4.17, 2.94, data not shown). Across both SDF and sealant/ART groups, average OHRQoL improved following treatment (Table 2): COHIP scores slightly improved from an average of 16.44 (SD = 11.12) in children receiving silver diamine fluoride at baseline to 14.62 (SD = 11.90) at follow-up, while those receiving sealants and atraumatic restorative treatments improved from 16.65 (SD = 10.56) to 16.47 (SD = 11.09). In subjects with measured OHRQoL at least 6 months post-treatment, there were no differences in OHRQoL by treatment group (Table 3) adjusting for baseline COHIP scores, the number of decayed teeth, and sociodemographic factors (B = − 1.13, 95% CI = − 4.23, 1.97).

Comparisons of the analytic sample at the follow-up visit indicate children receiving silver diamine fluoride are non-inferior to those receiving traditional glass ionomer sealants and ART on self-reported oral health-related quality of life (mean difference = 1.85, 95% CI = − 2.10, 5.80). The point estimate favors silver diamine fluoride however the confidence interval is below the non-inferiority margin. Additionally, there was no significant interaction as no treatment differences were found among only subjects with baseline caries.

**Table 3:** Regression coefficients, SDF versus Sealants + ART and covariates for OHRQoL (N = 160)

Variable	B	95% CI
SDF	− 1.13	− 4.23, 1.97
Baseline OHRQoL	0.68	0.47, 0.90
Total decay	0.33	− 1.47, 2.13

Models also adjusted for race and age

## Discussion

As arresting and preventive agents for dental caries, certain materials used in atraumatic restorative treatments and glass ionomer sealants can be visually imperceptible under casual observation. In contrast, application of silver diamine fluoride results in permanent black staining of dental decay and superficial staining of the oral mucosa. Notably, perceptions of self are affected by facial aesthetics, being previously observed in adolescents seeking orthodontic treatment and in children with preexisting orofacial anomalies [31, 32]. As over 25% of CariedAway participants had untreated decay at baseline with significantly lower OHRQoL than caries-free children, concerns regarding the aesthetic impact of SDF, despite demonstrated clinical and economic benefits [9, 33], may be justified.

Oral health-related quality of life slightly improved following treatment with either silver diamine fluoride or sealants/ART, and children receiving SDF exceeded the minimally important difference threshold of the COHIP-SF necessary for patient-centered clinically meaningful change [34]. Results further suggest SDF was non-inferior to children receiving ART/sealants with respect to impact on OHRQoL. These results are consistent with other studies of SDF in children reporting similar effects when compared to alternative treatments, such as ART, fluoride varnish, or placebo [22, 23, 25–27].

Clinical application of silver diamine fluoride in the CariedAway trial does not include anterior teeth; SDF is often applied to posterior teeth in order to mitigate impacts on facial aesthetics. In children aged 5–9 years, the global prevalence rate of caries in deciduous teeth exceeds 40% [1]. However, decay most often occurs in the occlusal surface of molars and pre-molars [35], thus a restriction to posterior application will still treat a majority of underlying disease. In previous studies of SDF, caregivers of children with untreated caries were more accepting of the staining effect when applied to posterior lesions, if the child had a history of behavioral issues when treated by a dentist, or if more invasive measures, such as anesthesia, would be required [36]. Anterior application of silver diamine fluoride may be acceptable for deciduous teeth due to expected exfoliation, but more aesthetically pleasing alternatives may be required for permanent anterior teeth in adolescents.

The focus of this analysis was on the potential short-term impact of SDF application on oral health-related quality of life, relative to more traditional non-surgical caries treatments. It may be the case that the staining effect of SDF, even when confined to posterior teeth, becomes more appreciated with longer rates of follow-up or when children progress into adolescence where facial aesthetics may be of greater concern. Additionally, as overall oral health-related quality of life has been shown to be responsive to the severity of dental caries [37], the long-term impact on OHRQoL following treatment with SDF may behave in a similar manner.

The early suspension of the CariedAway trial due to the impact of the COVID-19 pandemic meant that we were unable to obtain 6-month follow-up data for a substantial proportion of the baseline sample, and longitudinal observation beyond 6 months was not viable. As a result, only a subset of those initially enrolled and completing OHRQoL assessments were able to be analyzed. While our initial power calculations used a total enrollment of 396, which assumed an ICC of 0.10, our results show that the actual cluster correlation is considerably smaller. Compared to the full baseline sample, the analytic sample was similar in sex, the prevalence of untreated decay, and the average number of decayed teeth, however there was a greater proportion of subjects of Hispanic or black race/ethnicity. As our primary objective was on the associations between oral health, non-surgical treatment, and OHRQoL, we do not expect the racial/ethnic differences to be a concern. Regardless, this selection bias is still a risk to internal validity, and the reported associations should be interpreted with caution. Future research in CariedAway will seek to explore more moderate- to long-term change in OHRQoL, which would not be as negatively affected by the COVID-19 pandemic.

Silver diamine fluoride can be applied in significantly less time than atraumatic restorative treatments [27] and does not require the same degree of clinical training, suggesting that SDF is more efficient as a pragmatic treatment for caries. For example, some states authorize registered nurses to provide SDF under the supervision of a licensed general dentist. Additionally, the non-invasive nature of SDF as an arresting agent, combined with its secondary preventive effects, make it an attractive alternative to more traditional non-surgical interventions [38]. Our results suggest that children do not perceive any negative impacts on oral health-related quality of life approximately 6 months following application. These findings, combined with documented evidence of safety and clinical efficacy, further support the continued use of silver diamine fluoride.

## Data Availability

The datasets generated and/or analysed during the current study are not publicly available due to the active nature of the trial but are available from the corresponding author on reasonable request.
